# Restoring physiological levels of ascorbate slows tumor growth and moderates HIF-1 pathway activity in Gulo^−/−^ mice

**DOI:** 10.1002/cam4.349

**Published:** 2014-10-30

**Authors:** Elizabeth J Campbell, Margreet C M Vissers, Stephanie Bozonet, Arron Dyer, Bridget A Robinson, Gabi U Dachs

**Affiliations:** 1 Mackenzie Cancer Research Group, Department of Pathology, University of OtagoChristchurch, New Zealand; 2 Centre for Free Radical Research, Department of Pathology, University of OtagoChristchurch, New Zealand; 3 CARA Research Facilities, University of OtagoChristchurch, New Zealand; 4 Canterbury Regional Cancer and Haematology Service, Canterbury District Health BoardChristchurch, New Zealand; 5 Department of Medicine, University of OtagoChristchurch, New Zealand

**Keywords:** Gulonolactone oxidase, hypoxia-inducible factor 1, VEGF, vitamin C

## Abstract

Hypoxia-inducible factor-1 (HIF-1) governs cellular adaption to the hypoxic microenvironment and is associated with a proliferative, metastatic, and treatment-resistant tumor phenotype. HIF-1 levels and transcriptional activity are regulated by proline and asparagine hydroxylases, which require ascorbate as cofactor. Ascorbate supplementation reduced HIF-1 activation in vitro, but only limited data are available in relevant animal models. There is no information of the effect of physiological levels of ascorbate on HIF activity and tumor growth, which was measured in this study. C57BL/6 Gulo^−/−^ mice (a model of the human ascorbate dependency condition) were supplemented with 3300 mg/L, 330 mg/L, or 33 mg/L of ascorbate in their drinking water before and during subcutaneous tumor growth of B16-F10 melanoma or Lewis lung carcinoma (LL/2). Ascorbate levels in tumors increased significantly with elevated ascorbate intake and restoration of wild-type ascorbate levels led to a reduction in growth of B16-F10 (log phase *P* < 0.001) and LL/2 tumors (lag growth *P* < 0.001, log phase *P* < 0.05). Levels of HIF-1*α* protein in tumors decreased as dietary ascorbate supplementation increased for both tumor models (*P* < 0.001). Similarly, tumor ascorbate was inversely correlated with levels of the HIF-1 target proteins CA-IX, GLUT-1, and VEGF in both B16-F10 and LL/2 tumors (*P* < 0.05). The extent of necrosis was similar between ascorbate groups but varied between models (30% for B16-F10 and 21% for LL/2), indicating that ascorbate did not affect tumor hypoxia. Our data support the hypothesis that restoration of optimal intracellular ascorbate levels reduces tumor growth via moderation of HIF-1 pathway activity.

## Introduction

As a solid tumor grows, its rapid expansion leads to areas with poor vascularization and an inadequate blood supply that causes the development of local areas of hypoxia [reviewed in 1]. This initiates the activation of hypoxia-inducible factor 1 (HIF-1) [reviewed in 2,3], a stress-response transcription factor that controls the synthesis of hundreds of proteins that define the metabolism of tumors and affect the microenvironment. Many enzymes involved in glycolysis and glucose transport (glucose transporter 1, GLUT-1), and that control cellular pH (carbonic anhydrase IX, CA-IX), are upregulated. HIF-1 can be induced by metabolic disturbance in addition to hypoxia, and is linked to the phenomenon known as the Warburg effect, which describes the glycolytic dependency of tumors even in the presence of oxygen 4. HIF-1 also controls proteins that drive angiogenesis (vascular endothelial growth factor, VEGF), tumor invasion, and metastasis 2,3. A lack of HIF-1 activity results in reduced angiogenesis and slower tumor growth rates 1,5, whereas increased HIF-1 activity promotes resistance of cancer cells to treatment by chemo- and radiotherapy 6 and is associated with poor prognosis in many cancer types 7,8. Hence, HIF-1 activation is associated with a more aggressive tumor phenotype.

The activity of HIF-1 is under tight control via posttranslational modification of the alpha subunit by a group of enzymes collectively known as the HIF hydroxylases. Hydroxylation of proline residues 402 and 564 targets the protein to the proteasome for rapid degradation 9,10, and hydroxylation of asparagine 803 prevents formation of an active transcriptional complex 11. The enzymes responsible for these reactions, prolyl hydroxylase (PHD) 1, 2, and 3, and factor-inhibiting HIF (FIH; asparaginyl hydroxylase) belong to the family of 2-oxoglutarate dioxygenases that also hydroxylate collagen and numerous other substrates 12. Their activity is dependent on the supply of molecular oxygen, 2-oxoglutarate and Fe^2+^, and a lack of any of these factors will prevent enzyme activity, making these enzymes effective oxygen and metabolic sensors 12. Vitamin C (ascorbate) is a cofactor for these enzymes and enhances their activity in cell-free systems 13, possibly by maintaining the active site Fe in its reduced state 14. This role is exclusive to ascorbate, which is structurally specific for the hydroxylase active site 13,15, and the role cannot be fulfilled by other antioxidant molecules 16. Low cellular ascorbate levels have been shown to decrease activity of the hydroxylases and increase HIF-1 levels and downstream gene expression in cell culture 17–19. These observations led us to hypothesize that tumor ascorbate levels could influence the activity of HIF-1, and affect tumor growth and aggression (defined by adverse pathology, increased spread and poor survival).

We have measured HIF-1 protein levels, downstream gene products, and ascorbate in tumors and adjacent normal tissue from patients with endometrial and colorectal cancer 20,21. Ascorbate accumulation in tumor tissue decreased with increasing tumor grade, and low ascorbate levels in all grades of tumor were associated with higher HIF-1 activation, with elevated VEGF protein levels and with increased tumor size. In contrast, tumors with higher ascorbate levels had minimal HIF-1 protein and lower expression of downstream gene products 20,21. In addition, colorectal cancer patients with higher ascorbate concentrations in their tumors had longer disease-free survival compared to those with lower tumor ascorbate levels 21.

In these clinical studies, however, ascorbate levels were not manipulated and an intervention trial would be necessary to determine whether changing ascorbate intake could influence tumor ascorbate levels, and whether this would influence HIF-1 levels and tumor growth. Prior to undertaking studies in cancer patients, this needs to be tested in an animal model, where dietary ascorbate intake can be closely controlled.

In animal models, administration of ascorbate has been shown to reduce tumor formation and progression 22–24 and this was sometimes associated with the inhibition of HIF-1 activity 22,24. However, these studies were carried out in animals that synthesize their own ascorbate; plasma and tissue levels were never depleted and ascorbate administration resulted in levels above the physiological range. In this sense, these animals were not a good approximation of the human condition, where plasma levels are highly variable and absolutely dependent on dietary intake 25,26. Whether ascorbate plays a role in cancer has been widely debated, and there is considerable interest in the potential worth of pharmacologic doses administered via an intravenous route 27. However, there is minimal information on the effect of dietary ascorbate intake on tumor growth and development.

Hence, a model of the human ascorbate dependency condition was developed, the gulonolactone oxidase (Gulo) knockout mouse 28. These animals lack Gulo, the terminal enzyme in the synthesis pathway that is also lacking in humans, and rely entirely on dietary ascorbate 29. Previous studies compared ascorbate supplemented Gulo^−/−^ mice with animals where ascorbate was completely withdrawn for 4 weeks 30–32. Under these conditions, Gulo^−/−^ mice are severely scorbutic 28,29, thus confounding any conclusions drawn regarding the impact of ascorbate on cancer or HIF-activity 30–32. In addition, this reflects an extreme deficiency state rarely encountered in the human population in modern times. In these scorbutic animals, implanted tumors grew faster 31,32, but plasma erythropoietin levels were not increased 30, compared to healthy animals.

In contrast, our study investigated the effect of varied ascorbate intake within a relevant physiological range on the growth characteristics of tumor implants and on the activation of HIF-1 in the growing tumor, in Gulo^−/−^ mice. The aim was to test our hypothesis that increased tumor ascorbate could lead to downregulation of HIF-1, and that this would be associated with a reduced tumor growth rate.

## Methods

### Mouse model

C57BL6/J B6.129P2-Gulo^tm1Unc/Ucd^ mice were originally purchased from the Mutant Mouse Resource Center (MMRC, University of California at Davis) and fed standard mouse chow. Gulo^−/−^ mice were bred from homozygous C57BL/6 Gulo^−/−^adults in the Christchurch Animal facility and genotyped by PCR after weaning 29. To generate mice with three clinically relevant plasma and tissue ascorbate levels, populations were maintained on varied levels in the drinking water for 1 month prior to experimentation, and throughout tumor growth; low (33 mg/L), medium (330 mg/L), or optimum (3300 mg/L) with 10 *μ*mol ethylene diaminetetra acetic acid to stabilize ascorbate, changed twice weekly. One month is required to reach tissue steady state 29. Wild-type C57BL/6 mice, maintained on standard mouse chow and normal drinking water, were used in parallel as controls. Tumors were implanted into female mice aged 6–10 weeks. Ethical approval for the study was obtained from the University of Otago Animal Ethics Committee (C04/11), and animal welfare was monitored and maintained following international guidelines 33.

### Tumor models

Two C57BL/6 syngeneic tumors, B16-F10 melanoma and Lewis lung carcinoma, certified from the American Type Culture Collection (ATCC, passage 6–8) were injected subcutaneously (s.c.) at 1 × 10^6^ cells/mouse into the right flank 34. Tumor size was gauged by caliper measurements three times per week, up to a maximum tumor volume of ∽1000 mm^3^ (tumor volume = width^2^ × length × *π*/6). The animals were sacrificed by isoflurane (Baxter, Deerfield, IL) overdose and cervical dislocation, and the tumors excised, organs harvested, and blood collected. Tumors were weighed and divided in half, with one half immediately flash frozen, and the remainder fixed using formalin. Plasma, organs, and tumors were stored frozen at −80°C.

### Tissue homogenates

Frozen tissues were homogenized to a fine powder in liquid nitrogen using a chilled mortar and pestle. The tissue wet weight was measured and phosphate buffer (pH 7.4, Sigma Aldrich, Auckland, New Zealand) was added to make a homogeneous suspension 20**.** With tumors, care was taken to select viable samples and to avoid obvious necrotic or hemorrhagic tissue. The DNA content of the tissue was measured by the addition of propidium iodide (Sigma Aldrich) to standardize the specimens for cell content, as described 20.

### Ascorbate analysis

Ascorbate in the tissue homogenate was stabilized by the addition of perchloric acid (0.54 mol/L containing 50 mmol/L diethylene triamine pentaacetic acid, 1:1 volume) and centrifuged to pellet the protein (10,600 *g* for 5 min). The supernatant was analyzed by reverse-phase high-performance liquid chromatography (HPLC) with electrochemical detection (EC) as described previously [20,29**]** with a fresh standard curve of sodium-l-ascorbate (Sigma Aldrich) prepared for each run. Ascorbate concentrations in the mouse drinking water were similarly measured by HPLC-EC.

### Western blot analysis

Tissue homogenates were standardized to DNA in SDS-PAGE sample buffer, as described 20,21, and separated by PAGE on a 4–12% BisTris gradient SDS gel and blotted onto polyvinylidene difluoride membranes (Invitrogen, Carlsbad, CA). Each gel had the same positive control (20 *μ*g protein of 1% O_2_ hypoxia-treated Lewis Lung whole-cell lysate) to normalize the signal between blots. Membranes were probed with primary antibodies against mouse HIF-1*α* (1/800; R&D Systems, Minneapolis, MN), GLUT-1 (1/20000; Abcam, Cambridge, UK), CA-IX (1/800, R&D Systems), or *β*-actin (1/10000, R&D Systems), and with secondary anti-goat or anti-mouse horseradish peroxidase-conjugated antibodies (DAKO, North Sydney, Australia). Membranes were developed using Amersham™ ECL Plus Western blotting detection system (GE Healthcare, Auckland, New Zealand), and protein bands were detected using a digital gel-doc system (Uvitec, Cambridge, UK). Multiple exposure times were used to ensure that the signal was not saturated, and measurements were taken in the linear range. Proteins bands were quantified using Alliance 2.7 software (Uvitec) and the protein of interest normalized against the positive control following confirmation of equal loading using *β*-actin.

### VEGF ELISA

Mouse tumor VEGF protein levels were measured in undiluted samples prepared earlier for DNA analysis, using a Quantikine enzyme-linked immunosorbent assay (ELISA) (R&D Systems) according to the manufacturer's specifications.

### Histology

Formalin-fixed tumors were prepared for paraffin-embedded (FFPE) blocks, sectioned and stained by hematoxylin and eosin (H&E) to show tissue morphology. The percentage of necrosis was estimated on one central section per tumor, by two independent observers (EJC, GUD), blinded to the treatment.

### Statistical analyses

Data were analyzed using SPSS 19.0 (Chicago, IL) with the alpha level set to 0.05. The Kolmogorov–Smirnov test determined the distribution of each variable. Correlations were determined using Spearman's correlation coefficient. Linear regression analysis was used for tumor growth and HIF-1 pathway score analyses. The student's *t*-test determined differences in ascorbate levels. Pearson's correlation was used to assess the linear relationship between tumor ascorbate levels and the normalized protein expression of HIF-1*α* and its downstream pathway genes.

## Results

### Tissue ascorbate levels in response to dietary intervention

**Figure 1:**
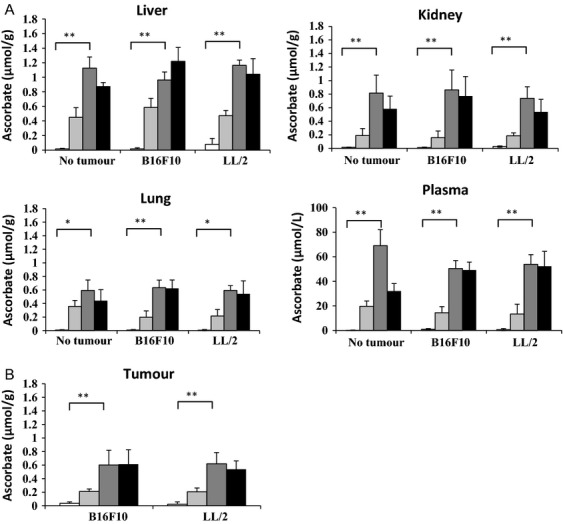
Determination of tissue ascorbate levels. Ascorbate concentration was measured by HPLC-ECD in liver, kidney, lung, tumor, and plasma from Gulo^−/−^ mice (shades of gray) or wild-type C57BL/6 mice (black), implanted with B16-F10 and LL/2 cells, or without tumors. Gulo^−/−^ mice were supplemented with ascorbate at 33 mg/L (light gray), 330 mg/L (mid gray), 3300 mg/L (dark gray) or unsupplemented for wild-type mice (black). Results are expressed as means ± SD; *n* = 10 per group for Gulo^−/−^ mice, *n* = 7–8 for wild-type mice; *P* < 0.001**. *P* < 0.05*. (A) Ascorbate concentrations in organs and plasma. (B) Ascorbate concentrations in tumors.

The ascorbate content of organ tissue and plasma from Gulo^−/−^ mice supplemented with three different physiological levels of ascorbate was measured, and levels were compared to those of wild-type mice (Fig.1A). There were considerable differences between levels in different organs, with higher ascorbate content in liver, and lesser levels in the kidney and lung, similar to our previous report 29.

The tissue levels reflected the dietary ascorbate supplementation, with the highest dose (3300 mg/L) resulting in tissue levels equivalent to those present in wild-type mice (Tables1 and 2). This dose was therefore considered to deliver optimal tissue ascorbate. The tissues of mice consuming drinking water with 330 mg/L ascorbate contained significantly less ascorbate than the wild-type animals, suggesting that this dose is suboptimal, as we noted previously 29. The Gulo^−/−^ mouse is normally maintained on this supplement dose, without apparent ill-health or weight loss 28,29. This was confirmed in this study with these animals being apparently healthy and maintaining or increasing body weight over the experimental period (mouse weights in Fig. S1). In contrast, the mice supplemented with low levels (33 mg/L) had very low levels of ascorbate in plasma and tissues (Fig.1A). Despite this, the animals could be maintained for 6–7 weeks without developing a complete deficiency phenotype, which is readily apparent within 3 weeks if ascorbate is completely withheld 28,29. Thereafter, some mice started to exhibit deficiency symptoms (malaise, lethargy, and weight loss), at which point experiments were terminated.

**Table 1:** Ascorbate concentrations of plasma and liver in Gulo^−/−^ mice, with and without a tumor burden, at three different levels of supplementation

Ascorbate supplement (mg/L)	33	*P*^2^	330	*P*^2^	3300	*P*^2^
Plasma (*μ*mol/L)^1^						
No tumor	0.14 ± 0.25		19.7 ± 4.6		69.0 ± 13.9	
B16-F10	0.68 ± 0.19	**<0.01**	13.4 ± 5.2	**0.01**	53.8 ± 6.9	**<0.01**
LL/2	0.86 ± 0.53	**<0.01**	14.5 ± 3.0	**<0.01**	50.4 ± 8.3	**<0.01**
Liver (*μ*mol/g)^1^						
No tumor	0.02 ± 0.00		0.45 ± 0.14		1.13 ± 0.33	
B16-F10	0.01 ± 0.00	0.99	0.47 ± 0.13	0.70	1.16 ± 0.17	0.07
LL/2	0.07 ± 0.03	**<0.01**	0.59 ± 0.07	**<0.01**	0.96 ± 0.20	0.21

1 Mean ± SD, *n* = 10 per group.

2 Comparing levels with and without tumor.

Significant values, (i.e. *P* < 0.5) are shown in bold.

**Table 2:** Ascorbate concentrations of plasma and organs from C57BL/6 wild-type mice with and without a tumor burden

	Tumor^1^	*n*	Ascorbate	*P* 2
Plasma (*μ*mol/L)	−	6	32.1 ± 6.1	**<0.01**
	+	9	51.2 ± 10.0	
Liver (*μ*mol/g)	−	6	0.87 ± 0.05	**0.02**
	+	9	1.2 ± 0.2	
Kidney (*μ*mol/g)	−	6	0.58 ± 0.19	0.29
	+	9	0.82 ± 0.25	
Lung (*μ*mol/g)	−	6	0.44 ± 0.16	0.07
	+	9	0.59 ± 0.30	

Mean ± SD.

1 Combined B16-F10 and LL/2 tumors.

2 Comparing levels with to without tumor.

### Tissue ascorbate levels in response to tumor load

It appeared that plasma and tissue ascorbate levels were affected by tumor burden in both the Gulo^−/−^ and wild-type mice. In the Gulo^−/−^ mice supplemented with 330 or 3300 mg ascorbate/L, the measured level of plasma ascorbate was significantly lower in those carrying a tumor burden than in those without a tumor burden (*P* ≤ 0.01, Table1). This suggests that there is increased turnover of ascorbate in the tumor-bearing animals. Ascorbate levels in mice supplemented with 33 mg/L were extremely low in all tissues and did not show this trend, nor did levels in the liver.

In contrast, in wild-type mice, the measured ascorbate levels in plasma and liver were significantly higher with a tumor burden (B16-F10 or LL/2), than without a tumor burden (plasma *P* = 0.001, liver *P* = 0.016; Table2). A similar trend was also evident in lung tissue (*P* = 0.07). This indicates that, in response to a tumor challenge, wild-type animals may compensate by increasing ascorbate production.

Tissue ascorbate levels in tumor-bearing wild-type mice were not different from tumor-bearing Gulo^−/−^ mice supplemented with 3300 mg/L ascorbate (plasma *P* = 0.48, liver *P* = 0.89), showing that optimal supplementation of Gulo^−/−^ mice restored tissue ascorbate levels to wild type.

### Tumor ascorbate levels in response to dietary intervention

Dietary intake also influenced tumor ascorbate levels (Fig.1B), with levels after supplementation with 3300 mg/L (optimal) resulting in three times as much, and more than 10 times as much, ascorbate being present in the tumor than when a dose of 330 mg/L and 33 mg/L was supplied, respectively. Supplementation at 3300 mg/L resulted in tumor levels of 0.61 ± 0.22 and 0.60 ± 0.17 *μ*mol/g, 330 mg/L resulted in levels of 0.20 ± 0.15 and 0.21 ± 0.06 *μ*mol/g, and 33 mg/L resulted in tumor levels of 0.02 ± 0.01 and 0.03 ± 0.02 *μ*mol/g, in B16-F10 and LL/2 tumors, respectively. These levels were comparable to those measured in the lung and kidney tissue and notably lower than levels measured in liver (Fig.1A and B). Ascorbate levels in tumors from Gulo^−/−^ mice supplemented with 3300 mg/L ascorbate were equivalent to the levels measured in tumors grown in wild-type mice (Fig.1B).

The tumor samples used to make homogenates constituted 2–5% of the entire tumor by weight. To assess how representative the homogenate sample was of the entire tumor, three additional random biopsies were taken from one B16-F10 and one LL/2 frozen tumor (330 mg/L ascorbate group). Ascorbate concentrations were similar to those found in the first sampling (B16-F10 original sample: 0.29 *μ*mol/g, subsequent samples: 0.43, 0.29, and 0.33 *μ*mol/g; LL/2 original sample: 0.23 *μ*mol/g, subsequent samples: 0.14, 0.29, 0.23 *μ*mol/g) demonstrating some tumor heterogeneity, but were not statistically different from the mean of the samples in the group (B16-F10 *P* = 0.36, LL/2 *P* = 0.53).

### Effect of ascorbate on tumor growth

**Figure 2:**
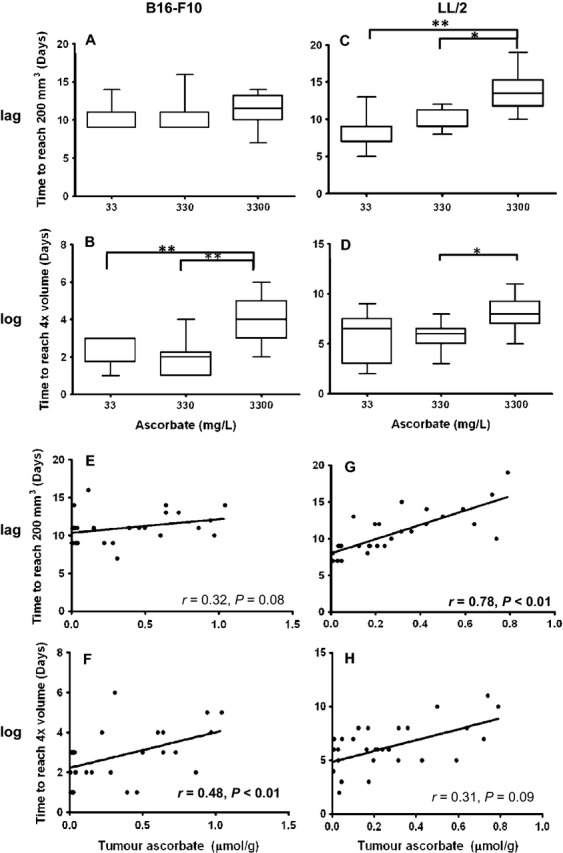
Tumor growth in Gulo^−/−^ mice. Boxplot of lag and log-phase growth of B16-F10 (A and B) and LL/2 (C and D) tumors grown in Gulo^−/−^ mice supplemented with either 3300 mg/L, 330 mg/L, or 33 mg/L ascorbate. Pearson's correlation between individual tumor ascorbate concentrations and lag phase growth in B16-F10 (E) and LL/2 (F) tumors, and log-phase growth in B16-F10 (F) and LL/2 (H) tumors; *n* = 10 per treatment group.

All Gulo^−/−^ mice implanted with B16-F10 and LL/2 cells (10^6^ cells/mouse implanted s.c.) developed tumors. Tumor growth rates, defined as lag phase (time from implantation to reach 200 mm^3^) and log phase (time to reach 4× volume, i.e., growth from 200 to 800 mm^3^) were influenced by ascorbate supplementation (Fig.2, data for individual mice are shown in Fig. S2).

When B16-F10 tumors were implanted, there was no difference in the lag phase (*P* = 0.496, Fig.2A), but these tumors took significantly longer to reach 4× volume in mice supplemented with optimal ascorbate (log phase: 3.9 ± 1.1 days) than in mice supplemented with either medium (2.0 ± 0.90 days) or low levels of ascorbate (2.4 ± 0.84 days) (*P* < 0.001) (Fig.2B).

In Gulo^−/−^ mice implanted with LL/2, optimal ascorbate supplementation significantly delayed initial tumor growth (lag phase: optimal 13.3 ± 2.6 days vs. medium 9.8 ± 1.39 days and low 9.1 ± 2.6 days, *P* < 0.001) (Fig.2C). During log-phase tumor growth, LL/2 grown in mice supplemented with optimal ascorbate also took significantly longer to reach 4× volume than mice supplemented with medium ascorbate (*P* < 0.05, Fig.2D).

Measured tumor ascorbate levels, as opposed to supplementation levels, also tended to correlate with lag phase tumor growth in B16-F10 (*P* = 0.08, Fig.2E), and in LL/2 tumors correlated strongly with lag phase growth (*P* < 0.001, by Pearson correlation) (Fig.2G). The more ascorbate accumulated in the tumors, the longer it took for tumors to initiate growth. Similarly, measured ascorbate in tumors correlated strongly with log-phase tumor growth for B16-F10 (*P* < 0.01, Fig.2F), and tended to correlate with log-phase growth in LL/2 tumors (*P* = 0.09) (Fig.2H).

### Effect of ascorbate on the HIF-1 pathway

**Figure 3:**
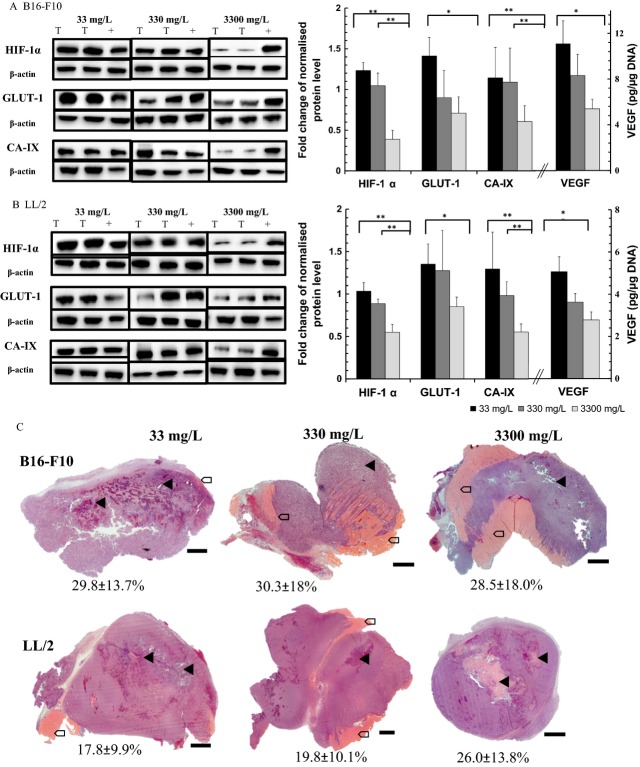
Protein levels of the HIF-1 pathway and necrosis in tumors. Protein levels of HIF-1 and its targets GLUT-1, CA-IX, and VEGF in (A) B16-F10 and (B) LL/2 tumors from Gulo^−/−^ mice supplemented with three levels of ascorbate. Examples of western blots show two lysates from tumors (T) and the positive control (+, LL/2 cells exposed to hypoxia) per panel. Densitometry analysis from western blots and VEGF ELISA results are expressed as mean ± SD. *P* < 0.001**. *P* < 0.05*. (C) Representative micrographs of H&E-stained tumors. B16-F10 and LL/2 tumors from Gulo^−/−^ mice supplemented with 33, 330, or 3300 mg/L of ascorbate. Mean percentage necrosis ± SD (*n* = 10 per group) are shown beneath each representative tumor. Areas of necrosis are indicated with arrow heads, invasion into muscle with arrows; scale bar = 500 *μ*m.

Levels of HIF-1*α* protein in B16-F10 and LL/2 tumors increased significantly as dietary ascorbate supplementation decreased (*P* < 0.001, Fig.3A and B). Significantly more HIF-1*α* protein was detected in tumors of mice supplemented with 33 or 330 mg/L ascorbate, compared to 3300 mg/L (*P* < 0.001). To determine the effect of ascorbate on HIF-1 transcriptional activity, the protein levels of its downstream targets (GLUT-1, CA-IX, and VEGF) were investigated. In parallel with HIF-1*α* protein levels, GLUT-1 levels decreased with increasing ascorbate supplementation in both tumor models (*P* < 0.05, Fig.3A and B). Similarly, CA-IX levels decreased with increasing supplementation (*P* < 0.05 for B16-F10 and *P* < 0.001 for LL/2). When mice were supplemented with optimal levels of ascorbate, their tumor VEGF levels were significantly reduced (2.8 ± 0.4 and 5.4 ± 2.5 pg/*μ*g DNA in B16-F10 and LL/2, respectively) compared to low ascorbate supplemented mice (5.6 ± 0.7 and 11.0 ± 4.6 pg/*μ*g DNA in B16-F10 and LL/2, respectively, both *P* < 0.05 using *t*-test).

When measured tumor ascorbate concentrations, rather than supplementation levels, were compared against HIF-1*α*, GLUT-1, CA-IX (from Western blot), and VEGF (from ELISA) protein levels, a strong and significant inverse association was detected (Table3). Higher tumor ascorbate levels correlated with low expression of all the HIF-1 pathway proteins measured.

**Table 3:** Correlation between tumor ascorbate concentrations and levels of HIF-1 pathway members

Tumor	*n*	Correlation	*P* 3
HIF-1*α*^1^			
B16-F10	30	−0.87	**<0.01**
LL/2	30	−0.83	**<0.01**
GLUT-1^1^			
B16-F10	30	−0.46	**0.04**
LL/2	30	−0.56	**<0.01**
CA-IX^1^			
B16-F10	30	−0.54	**<0.01**
LL/2	30	−0.71	**<0.01**
VEGF^2^			
B16-F10	28	−0.62	**<0.01**
LL/2	28	−0.49	**0.02**

1 Relative protein level according to densitometry reading (details in methods).

2 Protein levels measured by ELISA.

3 Significance, two-tailed.

### Effect of ascorbate on tumor pathology

B16-F10 tumors had a characteristic black appearance and soft, semiliquid morphology, whereas Lewis lung carcinomas appeared pale and more solid. Both tumor types tended to invade the underlying muscle of the back, irrespective of tumor size, and had macroscopic blood supply emanating from the skin. Large areas of necrosis were detected on H&E-stained sections of both tumor types (Fig.3C), as is expected in rapidly growing experimental tumors. There was no difference in the estimated area of necrosis seen in any of the tumors (Fig.3C); with a mean 30% necrosis for B16-F10 and 21% for LL/2. Ascorbate supplementation did not affect the level of necrosis (one-way analysis of variance (ANOVA), *P* = 0.44 for B16-F10 and *P* = 0.92 for LL/2). These data suggest that tumor ascorbate levels are not solely a reflection of necrosis, resulting from a poor blood supply.

Our previous study in clinical samples had shown an association between tumor size and tumor ascorbate levels 20,21. In Gulo^−/−^ mice, no association between levels of ascorbate in the tumor and tumor weight or size was evident in either B16-F10 (*P* = 0.38) or LL/2 models (*P* = 0.21, one-way ANOVA), as all mouse tumors were removed when they had reached a similar size.

## Discussion

This intervention study presents evidence, for the first time, that restoration of ascorbate from adequate to optimal physiological levels reduced tumor growth rates and moderated HIF-1 pathway activity in a mouse that models the human ascorbate dependency condition. Even a moderate decrease in tissue ascorbate content, to levels still considered adequate for normal health, had a significant impact on tumor pathology. These results provide an important insight into the human condition, as individual ascorbate levels vary considerably 35, and many cancer patients have lower plasma ascorbate levels than the healthy population 36–38.

In our study, we used physiologically relevant doses of ascorbate that were verified by measuring a range of tissue concentrations and comparing to wild-type levels. In this way, our study differed fundamentally from previous studies that used either wild-type mice, able to synthesize their own ascorbate 22,23, or that used Gulo^−/−^ mice under conditions of extreme deficiency 30–32. We detected significant changes between animals on the normal maintenance dose of ascorbate (330 mg/L) and animals on a dose which restored wild-type levels of ascorbate (3300 mg/L). Both melanoma and lung carcinoma models grew more slowly during log-phase and LL/2 tumors took longer to start growing, and tumor ascorbate levels correlated inversely with the expression of components of the HIF-1 pathway. That even a moderate decrease in plasma ascorbate levels significantly increased tumor growth was a surprise, and this finding is potentially of significance for the human condition, where many individuals do not have optimal ascorbate levels. This trend continued when mice on low-dose ascorbate (33 mg/L) were included, supporting the hypothesis that ascorbate availability can moderate HIF-1 activity and affect tumor growth.

Taken together, our results provide compelling data that support the hypothesis that ascorbate is a necessary component in the regulation of HIF-1 and that higher levels of tumor ascorbate are able to decrease HIF-1 activation and impact on tumor growth rates.

The use of wild-type control mice in our study has provided interesting insight into the potential need for ascorbate in managing a tumor burden. We measured higher plasma and liver ascorbate levels in wild-type mice bearing tumors, suggesting that these animals increased their ascorbate synthesis. Very early studies have suggested that animals increase vitamin C synthesis under stress conditions 39 and we hypothesize that wild-type mice increased ascorbate synthesis in response to tumor load; this observation has not been reported before. Our Gulo^−/−^ mouse data support the suggestion that a tumor burden results in increased ascorbate turnover, as plasma, liver, and lung ascorbate were lower in these animals than in Gulo^−/−^ mice without tumors. There is evidence that a similar phenomenon occurs in the human population: severely ill patients 40–42 and some cancer patients 36,37 have lower levels of plasma ascorbate than healthy people. This suggests that a cancer burden may require a higher ascorbate intake to maintain homeostasis, but further studies are required to determine the mechanisms involved in depletion.

It has been suggested that tumor ascorbate concentrations may merely reflect the abnormal tumor physiology (hypoxia or necrosis due to poor vascular supply). This study detected no difference in areas of tumor necrosis across the three ascorbate groups, despite significant differences in tumor ascorbate levels, tumor growth rates, and HIF-1 levels and activity. These findings suggest that tumor ascorbate is affected by factors other than tumor physiology. These are likely to include blood supply and could include the expression and activity of the vitamin C transporters 43. That tumor ascorbate was not simply a reflection of tumor physiology supports the argument for its playing an active role in determining tumor growth rate and progression.

We have previously carried out analyses on human tumor tissue and have reported an association between tumor ascorbate levels and HIF-1 activation and tumor size 20,21. However, manipulation of human tumor ascorbate has not (yet) been possible. This animal study has allowed us to more accurately control the ascorbate dosage and to monitor tumor growth conditions. Using this model, we have been able to demonstrate that tumor ascorbate levels correlated inversely with tumor growth and with levels of HIF-1, as well as its target proteins that control angiogenesis (VEGF), pH balance (CA-IX), and glucose transport (GLUT-1). These results therefore indicate that it is indeed conceivable that increased intracellular ascorbate was able to reduce tumor growth through its action as a cofactor for HIF hydroxylases 2,3. In addition to this activity, it is notable that ascorbate acts as cofactor for numerous metallo-enzymes, and could thereby also affect collagen synthesis 44 and epigenetic changes 45, among others, that could also impact on tumor growth rates. Indeed, we hypothesize that several molecular pathways that require ascorbate as cofactor may work in unison to control tumor growth and pathology.

The maintenance dose of ascorbate used in this study (330 mg/L) was recommended as being adequate for normal health 28, and is comparable to the human recommended daily intake (RDI, 45 mg/day in New Zealand, 60 mg/day in United Kingdom, and 75–90 mg/day in United States [reviewed in 46]). This dose is historically derived from the intake required to avoid the risk of developing scurvy but generally falls below what is needed for tissue saturation in a healthy individual (approx. 200 mg). Additional recommendations have proposed an intake of ascorbate that may result in plasma saturation levels and reduce the risk of chronic disease (200 mg/day) 47 (http://www.health.govt.nz/publication/nutrient-reference-values-australia-and-newzealand). The optimal dose in this study was designed to ensure tissue saturation levels as occurs in wild-type animals that synthesizes their own vitamin C, and our animal data support a beneficial effect in cancer at this dose.

There is considerable interest in the capacity of dietary ascorbate to act in a preventative capacity in addition to its proposed role at pharmacological doses in cancer treatment 46,48,49. This is the first intervention study to investigate the significance of restoring physiological ascorbate levels on tumor progression. Our data provide evidence in a suitable animal model that optimal ascorbate levels can reduce tumor growth and HIF-regulated cancer aggression.

## Conflict of Interest

None declared.

## Supporting Information

**Figure S1.** Percentage of body weight change over time in mice implanted with either B16-F10 melanomas or Lewis lung carcinoma at 1 × 10^6^ cells/mouse (*n* = 10 per treatment group). **Figure S2.** Tumor growth in individual Gulo^−/−^ mice implanted with B16-F10 melanoma and Lewis lung carcinoma, supplemented with 33, 330, and 3300 mg/L ascorbate in their drinking water (*n* = 10 per treatment group).
