# Supplementary material for: Restoring physiological levels of ascorbate slows tumor growth and moderates HIF-1 pathway activity in Gulo−/− mice
Source: Cancer Med. 2014 Oct 30;4(2):303–14. doi: 10.1002/cam4.349 (PMC4329013; doi:10.1002/cam4.349)
Supplement: Supplementary file 1 [file cam40004-0303-sd1.pptx]

## Slide 1
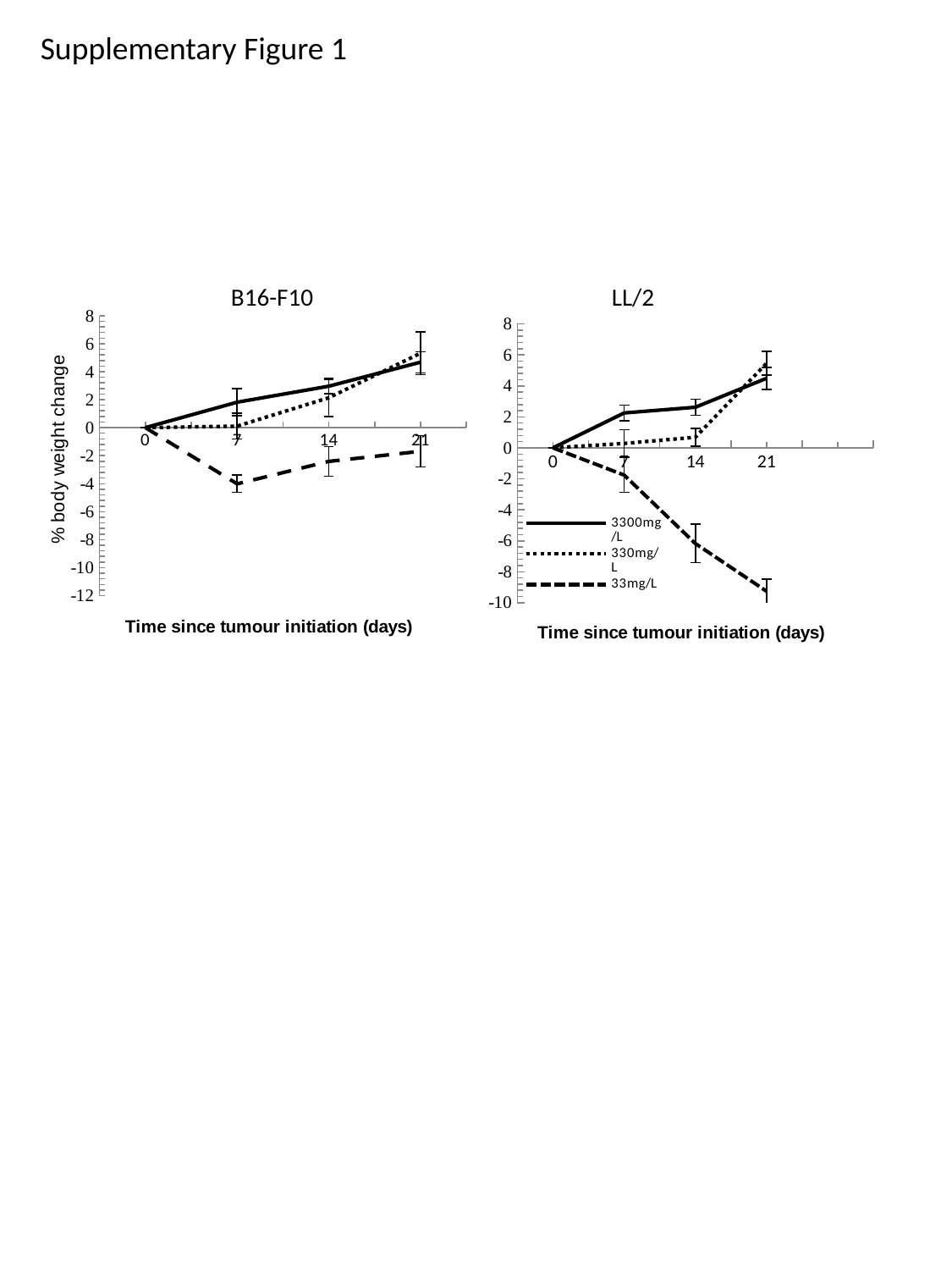

Supplementary Figure 1
B16-F10			LL/2
[unsupported chart]
### Chart
| Category | | | |
|---|---|---|---|
| 0 | 0.0 | 0.0 | 0.0 |
| 7 | 1.8184791300645646 | 0.10840907226639032 | -4.010683062282055 |
| 14 | 2.9608324192275504 | 2.137696087623131 | -2.407244149975446 |
| 21 | 4.6771539145781516 | 5.315276196776262 | -1.6883131008897443 |

## Slide 2
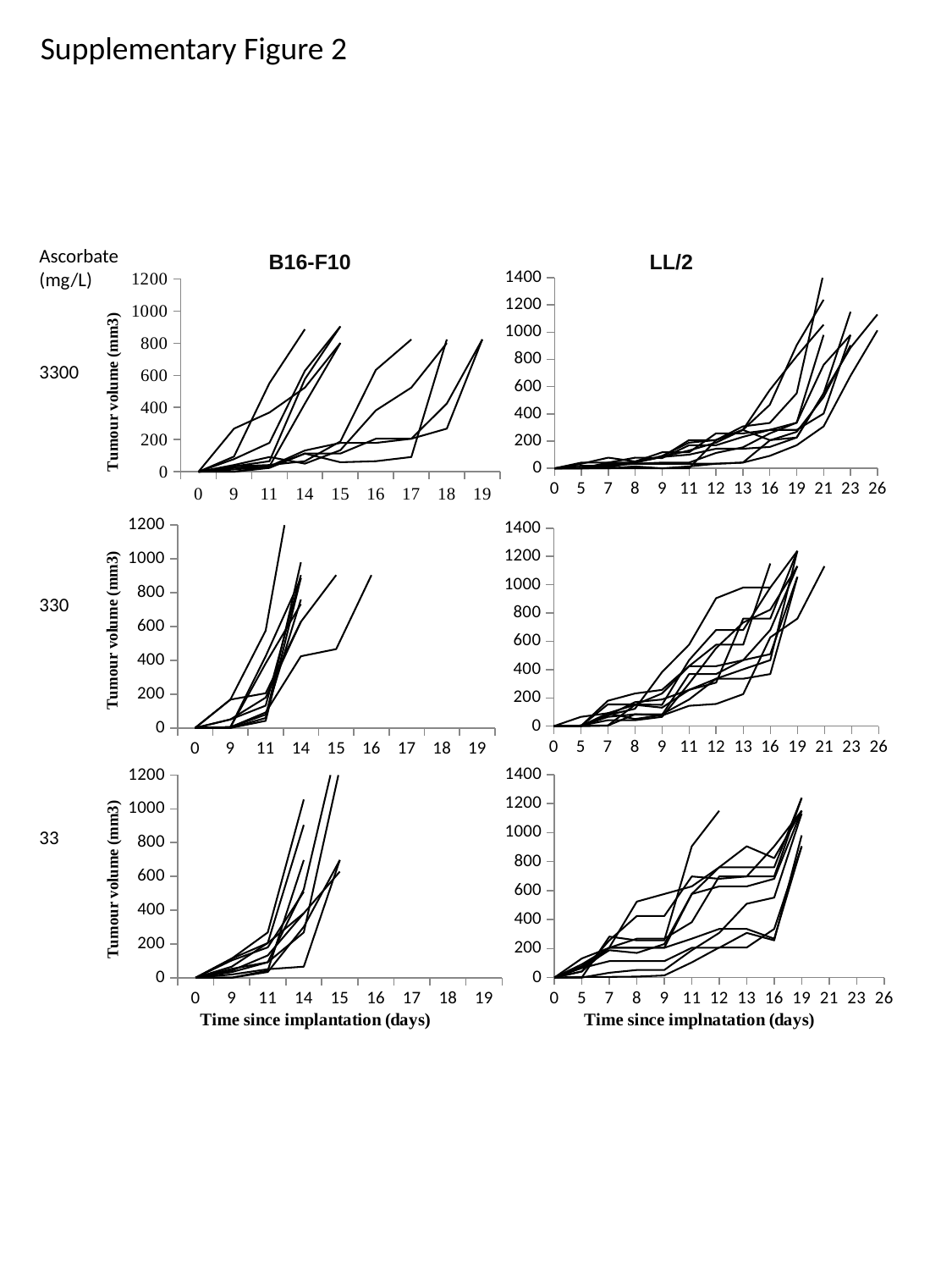

Supplementary Figure 2
Ascorbate
(mg/L)
3300
330
33
B16-F10			LL/2
[unsupported chart]
[unsupported chart]
[unsupported chart]
[unsupported chart]
[unsupported chart]
[unsupported chart]
